# Substitutions in SARS-CoV-2 Mpro Selected by Protease Inhibitor Boceprevir Confer Resistance to Nirmatrelvir

**DOI:** 10.3390/v15091970

**Published:** 2023-09-21

**Authors:** Karen Anbro Gammeltoft, Yuyong Zhou, Line Abildgaard Ryberg, Long V. Pham, Alekxander Binderup, Carlos Rene Duarte Hernandez, Anna Offersgaard, Ulrik Fahnøe, Günther Herbert Johannes Peters, Santseharay Ramirez, Jens Bukh, Judith Margarete Gottwein

**Affiliations:** 1 Copenhagen Hepatitis C Program (CO-HEP), Department of Infectious Diseases, Copenhagen University Hospital-Hvidovre, Kettegård Alle 30, 2650 Hvidovre, Denmark; karen.anbro.gammeltoft@regionh.dk (K.A.G.); yuyong.zhou@regionh.dk (Y.Z.); line.ryberg@sund.ku.dk (L.A.R.); pham@sund.ku.dk (L.V.P.); alekxander.marcus.binderup@regionh.dk (A.B.); carlos.rene.duarte.hernandez@regionh.dk (C.R.D.H.); anna.offersgaard@regionh.dk (A.O.); ulrik@sund.ku.dk (U.F.); santseharayra@sund.ku.dk (S.R.); jbukh@sund.ku.dk (J.B.); 2 Copenhagen Hepatitis C Program (CO-HEP), Department of Immunology and Microbiology, Faculty of Health and Medical Sciences, University of Copenhagen, Blegdamsvej 3B, 2200 Copenhagen, Denmark; 3 Department of Chemistry, Technical University of Denmark, 2800 Kongens Lyngby, Denmark; ghp@kemi.dtu.dk

**Keywords:** SARS-CoV-2, COVID-19, antiviral resistance, protease inhibitor, boceprevir, nirmatrelvir, Mpro, Mpro inhibitor

## Abstract

Nirmatrelvir, which targets the SARS-CoV-2 main protease (Mpro), is the first-in-line drug for prevention and treatment of severe COVID-19, and additional Mpro inhibitors are in development. However, the risk of resistance development threatens the future efficacy of such direct-acting antivirals. To gain knowledge on viral correlates of resistance to Mpro inhibitors, we selected resistant SARS-CoV-2 under treatment with the nirmatrelvir-related protease inhibitor boceprevir. SARS-CoV-2 selected during five escape experiments in VeroE6 cells showed cross-resistance to nirmatrelvir with up to 7.3-fold increased half-maximal effective concentration compared to original SARS-CoV-2, determined in concentration–response experiments. Sequence analysis revealed that escape viruses harbored Mpro substitutions L50F and A173V. For reverse genetic studies, these substitutions were introduced into a cell-culture-infectious SARS-CoV-2 clone. Infectivity titration and analysis of genetic stability of cell-culture-derived engineered SARS-CoV-2 mutants showed that L50F rescued the fitness cost conferred by A173V. In the concentration–response experiments, A173V was the main driver of resistance to boceprevir and nirmatrelvir. Structural analysis of Mpro suggested that A173V can cause resistance by making boceprevir and nirmatrelvir binding less favorable. This study contributes to a comprehensive overview of the resistance profile of the first-in-line COVID-19 treatment nirmatrelvir and can thus inform population monitoring and contribute to pandemic preparedness.

## 1. Introduction

Since the identification of severe acute respiratory syndrome coronavirus 2 (SARS-CoV-2) in 2019, the coronavirus disease 2019 (COVID-19) has become a major global public health threat. As of August 2023, there have been more than 770 million registered infections, posing a major socioeconomic burden. Reports from the World Health Organization (WHO) estimate that there was excess mortality in 2020 and 2021, with up to 16.6 million deaths attributed to COVID-19 [1,2]. Even though intense efforts led to the fast development of efficient prophylactic vaccines, treatment options for COVID-19 are still limited. Until recently, such treatments have primarily focused on alleviating symptoms or modulating the host’s immune system [3,4]. In contrast, there are only few approved drugs directly targeting SARS-CoV-2 proteins [5]. Such direct-acting antivirals include monoclonal antibodies that target the SARS-CoV-2 spike protein. At present, monoclonal antibodies have become inefficient and are no longer recommended due to currently circulating variants of concern [6,7]. At present, The United States Food and Drug Administration (FDA) has authorized three direct-acting antiviral therapeutics for treatment of COVID-19: (i) the polymerase inhibitor remdesivir (Veklury^®^, Gilead Sciences, Inc., Foster City, CA, USA), (ii) the polymerase inhibitor molnupiravir (Lagevrio^®^, Merck Sharp & Dohme LLC, Rahway, NJ, USA), and (iii) the main protease (Mpro) inhibitor nirmatrelvir (Paxlovid^®^, Pfizer, Inc., New York, NY, USA) [8]. Remdesivir, the first drug approved for COVID-19 treatment by both the FDA and the European Medicines Agency (EMA), is a nucleoside analog that targets the SARS-CoV-2 polymerase; it functions by template-dependent inhibition and by delay of RNA chain termination [9,10,11,12]. In clinical trials, treatment with remdesivir reduced the risk of hospitalization or death by 87% [13]. Nevertheless, the intravenous administration of remdesivir necessitates substantial resources [14]. The oral drug molnupiravir is a nucleoside analog that induces mutagenesis in the viral RNA genome. Clinical trials demonstrated a 31% reduction in the risk of hospitalization or death with molnupiravir treatment [15]. While it initially received emergency authorization from the FDA and EMA, it later failed to secure full authorization from the EMA due to its limited clinical efficacy [16,17,18,19]. The oral SARS-CoV-2 Mpro inhibitor nirmatrelvir has been approved for use by both the FDA and EMA and is currently the first-in-line COVID-19 treatment [20,21,22,23]. Nirmatrelvir has in clinical trials been shown to reduce the risk of COVID-19-associated hospitalization or death by 89% [24]. Further, nirmatrelvir is being considered for treatment of long-term effects of COVID-19 [25]. Additional Mpro inhibitors are in preclinical and clinical development, with some being in advanced clinical trials, such as ensitrelvir. Ensitrelvir, which has been approved in Japan, is now under Fast Track designation for COVID-19 treatment by the FDA [26,27,28]. Clinical trials demonstrated rapid viral clearance in patients with mild to moderate COVID-19 during treatment with ensitrelvir [29].

Because of the high mutation rate during RNA virus replication, it is expected that treatment of patients with direct-acting antivirals may give rise to treatment-resistant SARS-CoV-2 variants [30,31,32]. Further, there are case reports on patients failing treatment with nirmatrelvir [33,34,35]. Initial proof-of-concept studies have reported substitutions associated with resistance to currently authorized direct-acting antivirals including nirmatrelvir. Most of these studies were based on in vitro resistance selection and the study of naturally occurring polymorphisms in the drug targets [36,37,38,39,40,41,42,43,44]. In these studies, for nirmatrelvir, key resistance-associated substitutions (RAS) were found to be localized to Mpro position 166 [36,37,38,39,44]. Importantly, changes at Mpro position 166 were also observed in COVID-19 patients who were treated with nirmatrelvir, including one patient with treatment failure, highlighting the clinical relevance of the above-mentioned in vitro studies [45,46]. However, mechanisms leading to treatment failure remain to be studied, and a comprehensive overview of nirmatrelvir RAS is needed.

In previous drug-repurposing studies, we and others found that the hepatitis C virus (HCV) NS3 protease inhibitor boceprevir was active against SARS-CoV-2 by targeting Mpro [47,48,49]. However, the efficacy of boceprevir against SARS-CoV-2 is limited, and therefore, improved Mpro inhibitors have been developed. These efforts have led to the development of compounds, such as nirmatrelvir, that are structurally related to boceprevir but show improved activity against SARS-CoV-2 [50,51,52]. Here, we report boceprevir escape viruses with RAS in Mpro that had high fitness and conferred cross-resistance to nirmatrelvir in an infectious cell culture system. These findings will contribute to a more comprehensive overview of SARS-CoV-2 genetic correlates of resistance to nirmatrelvir, the first-in-line COVID-19 treatment.

## 2. Materials and Methods

### 2.1. Inhibitors, Cells, and Viruses

Boceprevir and nirmatrelvir were synthesized by Acme Bioscience (Palo Alto, CA, USA) and dissolved and stored as previously described [47].

All cell culture experiments were carried out in African green monkey kidney VeroE6 cells kindly provided by J. Dubuisson and human lung carcinoma A549-hACE2 cells (InvivoGen, Toulouse, France), which were maintained as previously described [36,47,53].

The virus stock of the so-called original SARS-CoV-2 was derived from the patient isolate SARS-CoV-2/human/Denmark/DK-AHH1/2020 (GenBank: MZ049597), representing an originally circulating variant with the D614G substitution in the spike protein, and was generated as described previously [54]. Stocks of the polyclonal boceprevir escape viruses were obtained as described in “Selection for SARS-CoV-2 Resistance to Boceprevir”. Stocks of the recombinant SARS-CoV-2 mutants were obtained as described in “Generation of Recombinant SARS-CoV-2 Mutants”. All infectious cell culture experiments were carried out under biosafety conditions in accordance with Danish regulations and with permission from the Danish authorities.

### 2.2. Selection for SARS-CoV-2 Resistance to Boceprevir

To select for SARS-CoV-2 resistance to boceprevir, the original SARS-CoV-2 was passaged in the presence of increasing concentrations of boceprevir as previously described for nirmatrelvir [36]. To initiate the passaging, VeroE6 cells were seeded in T25 flasks (Thermo Fisher Scientific, Roskilde, Denmark) at a density of 1 × 10^6^ cells per flask, and both virus at 0.00002 multiplicity of infection (MOI) and specified concentrations of inhibitor were added the following day. Cell culture supernatants were collected every 48–72 h and stored at −80 °C; to maintain a subconfluent monolayer, cells were split, and the supernatant was replaced with fresh inhibitor containing medium. Upon splitting of cells, duplicate cultures were plated on chamber slides for immunostaining as described in “Immunostaining of Chamber Slides”. Five independent boceprevir escape experiments were carried out as specified. Selected supernatants were collected at the peak of infection and recovered viruses were subjected to next-generation sequencing (NGS). Polyclonal boceprevir escape virus 1 and 2 (BOC-EV1 and BOC-EV2) stocks were generated by inoculating 3 × 10^6^ VeroE6 cells, plated the previous day in T80 flasks, with 15 µL supernatant derived from Day 74 from escape 1 or Day 57 from escape 2. Upon splitting of cells, duplicate cultures were plated on chamber slides for immunostaining as described in “Immunostaining of Chamber Slides”. Cell culture supernatants were harvested at peak of infection and stored at −80 °C. Polyclonal escape viruses were used for short-term concentration–response and longer-term treatments.

### 2.3. Generation of Recombinant SARS-CoV-2 Mutants

A bacterial artificial chromosome (BAC) based reverse genetics system [55] was used to generate SARS-CoV-2 recombinants harboring Mpro mutations. These mutants were engineered using infusion cloning and megaprimer-based cloning.

In vitro transcriptions and transfections using these constructs were carried out as previously described [36,55]. In brief, for in vitro transcriptions, the mMESSAGE mMACHINE T7 Transcription Kit (Thermo Fisher, Waltham, MA, USA) was used. The RNA transcripts were then transfected into VeroE6 cells using Lipofectamine 2000 (Thermo Fisher Scientific). A 1 µg amount of in vitro RNA transcripts was used for the L50F, A173V, and L50F + A173V recombinants, and 2 µg was used for the C160F, A191V, L50F + C160F + A173V, L50F + A173V + A191V and the original recombinants. Cell culture supernatants were collected on Day 2 and Day 3 post-transfection. A 250 µL volume of supernatant was then used to infect VeroE6 cells in T80 flasks to produce virus stocks of the recombinant SARS-CoV-2 mutants. Upon splitting of cells, duplicate cultures were plated on chamber slides for immunostaining as described in “Immunostaining of Chamber Slides”. Cell culture supernatants were harvested at the peak of infection and stored at −80 °C. Virus stocks were used for short-term concentration–response treatments, longer-term treatments, and viral fitness experiments.

### 2.4. Antiviral Short-Term Concentration-Response Treatments

Short-term concentration-response treatments were carried out as previously described [36,47,53]. In brief, on Day −1, VeroE6 or A549-hACE2 cells were seeded at 10,000 cells per well in 96-well plates (Thermo Fisher Scientific, Roskilde, Denmark). On Day 0, cells were incubated with original SARS-CoV-2 as a control, specified polyclonal SARS-CoV-2 escape viruses, or recombinant mutants. Following 60 min incubation, the specified serially diluted inhibitors were added. All dilutions were tested in 4 or 7 replicates. Each treatment experiment included a non-treated infected control in 8 or 14 replicates and a non-treated non-infected control in 8 or 12 replicates. The original SARS-CoV-2 was included in each treatment experiment as a reference. On Day 2 post-infection (DPI) and treatment, the cells were fixed and immunostained as described in “Immunostaining of 96-Well Plates”. The % residual infectivity was calculated as counts of individual treated infected wells related to mean counts of non-treated infected wells. The 50% effective concentration (EC50) values were determined using GraphPad Prism 8.0.0 and applying the formula Y = Top/(1 + 10^(Log_10_EC50−X)∗HillSlope^). Log_10_(EC50) and log_10_SEM(EC50) (SEM, standard error of the mean) derived from replicate experiments were used to determine *p*-values by Z-test, as previously described [56]. *p*-values below 0.0001 were considered significant.

### 2.5. Immunostaining of 96-Well Plates

Following short-term concentration–response treatments, 96-well plates were fixed with methanol and stained with first antibody SARS-CoV-2 spike chimeric monoclonal antibody (Sino Biological #40150-D004, Beijing, China) at 1:5000 dilution and second antibody F(ab’)2-Goat anti-human IgG Fc Cross-Adsorbed Secondary Antibody, HRP (Invitrogen #A24476, Carlsbad, CA, USA) or Goat F(ab’)2 Anti-Human IgG—Fc (HRP), preabsorbed (Abcam #ab98595, Cambridge, UK) at 1:2000 dilution. Next, infected cells were visualized by staining with DAB substrate, BrightDAB kit (Immunologic#BS04-110, Duiven, The Netherlands). The ImmunoSpot Series 5 UV Analyzer (CTL Europe GmbH, Bonn, Germany) was used to evaluate the number of single SARS-CoV-2 spike-positive cells per well. Representative images of stained 96-well plates are shown in a previous publication [57].

### 2.6. Longer-Term Antiviral Treatments

Longer-term treatments were carried out in VeroE6 cells. In brief, on Day −1, VeroE6 cells were seeded at 1 × 10^6^ cells per flask in T25 flasks. On Day 0, specified polyclonal SARS-CoV-2 escape viruses, recombinant mutants, or original SARS-CoV-2 as a control were added at 0.00002 MOI together with the specified inhibitors. On Days 1, 3, and 5, cells were split, and supernatants were replaced with fresh inhibitor containing medium. Upon splitting of cells, cell culture supernatants were collected and stored at −80 °C for subsequent quantification of viral RNA, and duplicate cultures were plated on chamber slides for immunostaining as described in “Immunostaining of Chamber Slides”.

### 2.7. Immunostaining of Chamber Slides

During and following longer-term treatment assays, chamber slides were fixed with methanol and stained with first antibody SARS-CoV-2 spike chimeric monoclonal antibody (Sino Biological #40150-D004, Beijing, China) at 1:1000 dilution and second antibody Alexa Fluor 488 goat anti-human immunoglobulin G (IgG) (H + L) (Invitrogen #A-11013, Paisley, UK) at 1:500 dilution combined with Hoechst 33342 (Invitrogen, Paisley, UK) at 1:1000 dilution. Fluorescence microscopy (ZEISS Axio Vert.A1, Jena, Germany) was used to evaluate the percentages of SARS-CoV-2 spike-positive cells, using the following designations: 0% positive cells (no cells infected), single positive cells, and 10 to 90% positive cells, in steps of 10%.

### 2.8. Determination of SARS-CoV-2 RNA Titers

Assays were carried out as previously reported [36,47,58]. In brief, cell culture supernatants from longer-term treatments were extracted using Trizol LS Reagent (Life Technologies, Carlsbad, CA, USA) and chloroform (Sigma, Saint Louis, MI, USA). SARS-CoV-2 RNA was purified using the Zymo RNA Clean and Concentrator-5 kit (ZymoResearch, Irvine, CA, USA) for RT-qPCR according to the manufacturer’s protocol. Viral RNA was quantified by RT-qPCR on the LifeCycler 96 System (Roche) using the TaqMan Fast Virus 1-Step Master Mix (Thermo Fisher). For each sample, the RNA titer is given as a mean of two technical replicates. The lower limit of quantification (LLOQ) of the assay was determined as: (mean of RNA titers in non-infected control culture supernatants) + (3 standard deviations).

### 2.9. Determination of SARS-CoV-2 Infectivity Titers

Assays were carried out as previously reported [36,58]. In brief, 10,000 VeroE6 cells per well were seeded in 96-well plates on Day −1, and cells were infected with 10-fold serially diluted cell culture supernatants on Day 0 in 4 replicate cultures. On Day 3, cells were fixed and immunostained as described in “Immunostaining of 96-Well Plates”. SARS-CoV-2 infectivity titers were determined as 50% tissue culture-infectious dose per mL (TCID50/mL) using the Reed-Muench method. TCID50/mL titers are given as a means with SEM calculated from three replicate experiments with four technical replicates in each experiment. The lower limit of detection (LLOD) was 2 log_10_ TCID50/mL.

### 2.10. Evaluation of Genetic Stability of Recombinant SARS-CoV-2 Mutants

To evaluate their genetic stability, recombinant SARS-CoV-2 mutants were passaged four times in VeroE6 cells without inhibitor as previously described [36]. In brief, VeroE6 cells were seeded in T25 flasks at a density of 1 × 10^6^ cells per flask on Day −1. Cells were infected with 250 µL supernatant derived at the peak of infection from the previous culture on Day 0. From Day 1, to maintain a subconfluent monolayer, cells were split, and the supernatant was replaced with fresh medium from Day 1. Upon splitting of cells, duplicate cultures were plated on chamber slides for immunostaining as described in “Immunostaining of Chamber Slides”. Viruses recovered from the fourth passage culture supernatants collected at the peak of infection were subjected to NGS analysis.

### 2.11. Next-Generation Sequencing of SARS-CoV-2 Genomes

The SARS-CoV-2 RNA was extracted from virus containing cell culture supernatant as described above; RT-PCR was used to generate five amplicons and the NEBNext Ultra II FS DNA Library Prep kit (New England BioLabs, Ipswich, MA, USA) was used for library preparations [54]. NGS analysis was done as previously described [36,47,53,54,55]. Linkage analysis of Mpro substitutions was done as previously described [32].

### 2.12. Analysis of Cell Viability by the MTS Assay in VeroE6 and A549-hACE2 Cells

To confirm that applied concentrations of inhibitors were not cytotoxic, cell viability was evaluated using the CellTiter 96 Aqueous One Solution Cell Proliferation Assay (Promega, Madison, WI, USA) as previously described [36,47,53,57]. Inhibitor concentrations were tested in 3 replicates including a non-treated control in 12 replicates.

### 2.13. Structural Analysis of the Effect of the L50F and A173V Substitutions in the Mpro-boceprevir Structure

An X-ray crystallography structure of the SARS-CoV-2 Mpro with boceprevir bound was obtained from the Protein Data Bank (PDB) with PDB entry 7k40 [59]. The PyMOL Molecular Graphics System 2.5.0. Schrödinger, LLC was used for manipulations, analysis, and visualization of the structure. More specifically, we carried out in silico mutagenesis for mutating A173 to V173 and chose the best-fitting rotamer from a library of backbone-dependent rotamers. We used the PyMOL distance tool for visualizing hydrogen bonds between boceprevir and amino acids in the Mpro structure, and the PyMOL plugin shows bumps for visualizing steric clashes in the structure.

## 3. Results

### 3.1. Viral Escape from Boceprevir Treatment

We previously characterized the efficacy of a panel of protease inhibitors originally developed for treatment of chronic HCV infection against SARS-CoV-2 in VeroE6 and A549-hACE2 cells [47]. Compared to the other tested protease inhibitors, boceprevir, structurally related to the current first-in-line SARS-CoV-2 Mpro inhibitor nirmatrelvir, had a favorable selectivity index (SI=half maximal cytotoxic concentration (CC50)/EC50). This facilitated SARS-CoV-2 escape experiments in VeroE6 cells to identify substitutions associated with Mpro inhibitor resistance (Supplementary Table S1). During such escape experiments, we observed that the original SARS-CoV-2 was suppressed by 3-fold EC50 boceprevir. However, boceprevir escape viruses could overcome up to 7-fold EC50 boceprevir. Higher boceprevir concentrations could not be applied due to cytotoxicity [47]. Sequence analysis revealed that polyclonal escape viruses from 4 of 5 independent escape experiments acquired a combination of the substitutions L50F and A173V in Mpro in virtually all viral genomes, while the fifth only acquired A173V in 78% of the genomes of the viral population (Supplementary Table S1). Thus, viral escape from boceprevir was linked to acquisition of Mpro substitutions warranting further investigation.

### 3.2. Sensitivity of Polyclonal Boceprevir Escape Viruses to Boceprevir and Nirmatrelvir

**Figure 1:**
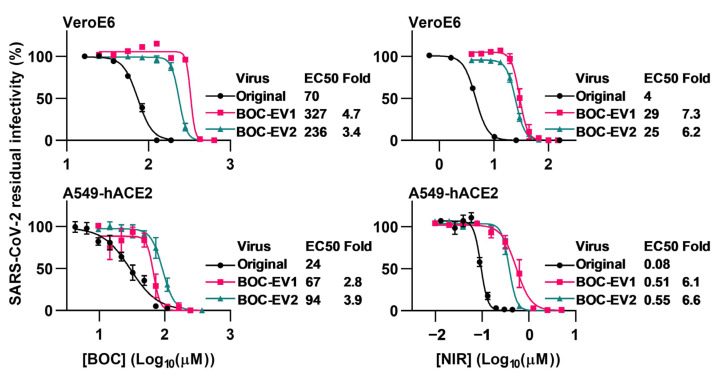
Short-term concentration–response treatments of polyclonal SARS-CoV-2 boceprevir escape viruses with boceprevir and nirmatrelvir. VeroE6 or A549-hACE2 cells, that were infected with boceprevir escape virus 1 (BOC-EV1), boceprevir escape virus 2 (BOC-EV2), or original SARS-CoV-2 and that were treated with specified concentrations of boceprevir (BOC) or nirmatrelvir (NIR) were visualized by immunostaining of spike protein and counted automatically. Data points represent residual infectivity determined as count of infected cells in treated infected wells relative to the mean of counts in non-treated infected control wells. Data points are given as means of 4 or 7 replicates with SEM. Representative curves and 50% effective concentration (EC50) values from replicate experiments were determined in GraphPad Prism 8.0.0. In the figure, one representative curve is shown, while EC50 values are median EC50 values calculated based on 1 to 13 replicate experiments. Fold resistance values were calculated as EC50_Escape Virus_/EC50_Original_. Median EC50 values, fold resistance values, and *p*-values are summarized in Supplementary Table S2. For the original SARS-CoV-2 treated with nirmatrelvir, the curves have been presented in a previous publication, while median EC50 values have been calculated in a different manner [36].

**Figure 2:**
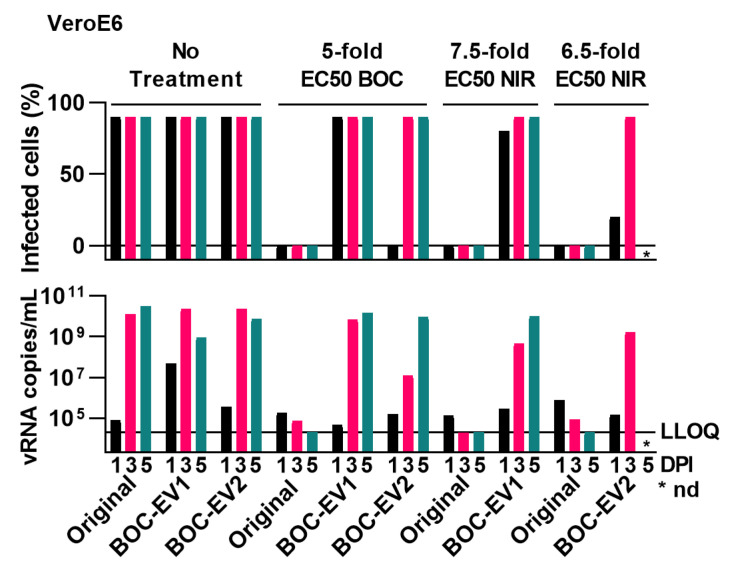
Longer-term treatment of polyclonal escape viruses with boceprevir and nirmatrelvir. VeroE6 cells that were infected with the original SARS-CoV-2 and the specified polyclonal boceprevir escape viruses (BOC-EV1 and BOC-EV2) were treated longer term with the specified fold-EC50 of boceprevir (BOC) or nirmatrelvir (NIR). % SARS-CoV-2 infected culture cells were determined by immunostaining of spike protein relative to immunostaining of nuclei on the specified days post-infection (DPI). Viral RNA (vRNA) titers in culture supernatants on the specified DPI were determined by RT-qPCR. LLOQ, lower limit of quantification. nd, not determined as culture was terminated due to virus induced cell death.

Compared to the original virus, selected polyclonal escape viruses with Mpro substitutions L50F and A173V, in the following termed BOC-EV1 and BOC-EV2, showed up to 4.7-fold increased EC50 (*p* < 0.0001) compared to the original virus in short-term concentration–response treatments with boceprevir (Figure 1, Supplementary Table S2). In addition, resistance was confirmed in longer-term treatments where BOC-EV1 and BOC-EV2 could overcome 5-fold EC50 of boceprevir, while the original virus was fully suppressed with no infected cells observed and viral RNA titers close to or below the LLOQ (Figure 2). Importantly, when testing these polyclonal escape viruses for nirmatrelvir cross-resistance, we found that they had up to 7.3-decreased nirmatrelvir sensitivity (*p* < 0.0001) compared to the original virus in VeroE6 and A549-hACE2 cells (Figure 1, Supplementary Table S2). The observed difference in nirmatrelvir potency between the two cell types can be attributed to the inherent expression of an efflux transporter by the VeroE6 cells, leading nirmatrelvir to be effectively transported out of the cells [60]. We additionally confirmed nirmatrelvir cross-resistance in longer-term treatments, where BOC-EV1 and BOC-EV2 could overcome up to 7.5- and 6.5-fold EC50 of nirmatrelvir, respectively (Figure 2), while the original virus was fully suppressed. Thus, polyclonal escape viruses were resistant to boceprevir and showed cross-resistance to nirmatrelvir, the first-in-line drug for treatment of COVID-19.

### 3.3. Sensitivity of Mutants with Engineered Mpro Substitutions to Boceprevir and Nirmatrelvir

**Figure 3:**
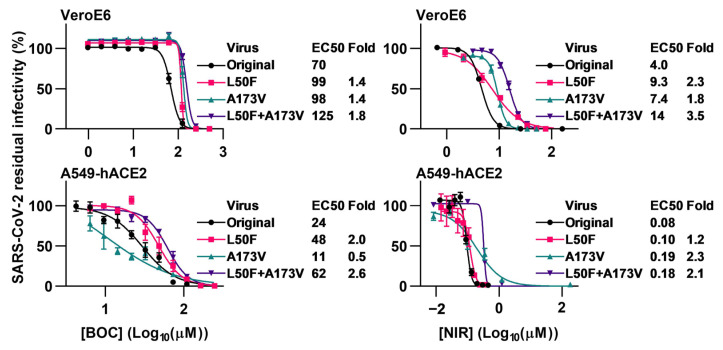
Short-term concentration–response treatments of engineered SARS-CoV-2 mutants with boceprevir and nirmatrelvir. VeroE6 cells or A549-hACE2 cells that were infected with SARS-CoV-2 engineered with specified RAS or original SARS-CoV-2 and that were treated with specified concentrations of boceprevir (BOC) or nirmatrelvir (NIR) were visualized by immunostaining of spike protein and counted automatically. Data points represent residual infectivity determined as count of infected cells in treated infected wells relative to the mean of counts of non-treated infected control wells. Data points are given as means of 4 or 7 replicates with SEM. Representative curves and 50% effective concentration (EC50) values from replicate experiments were determined in GraphPad Prism 8.0.0. In the figure, one representative curve is shown, while EC50 values are median EC50 values calculated based on 1 to 13 replicate experiments. Fold resistance values were calculated as EC50_Mutant_/EC50_Original_. Median EC50 values, fold resistance values, and *p*-values are summarized in Supplementary Table S2. For the original SARS-CoV-2 and the L50F mutant treated with nirmatrelvir, the curves have been presented in a previous publication, while median EC50 values have been calculated in a different manner [36].

**Figure 4:**
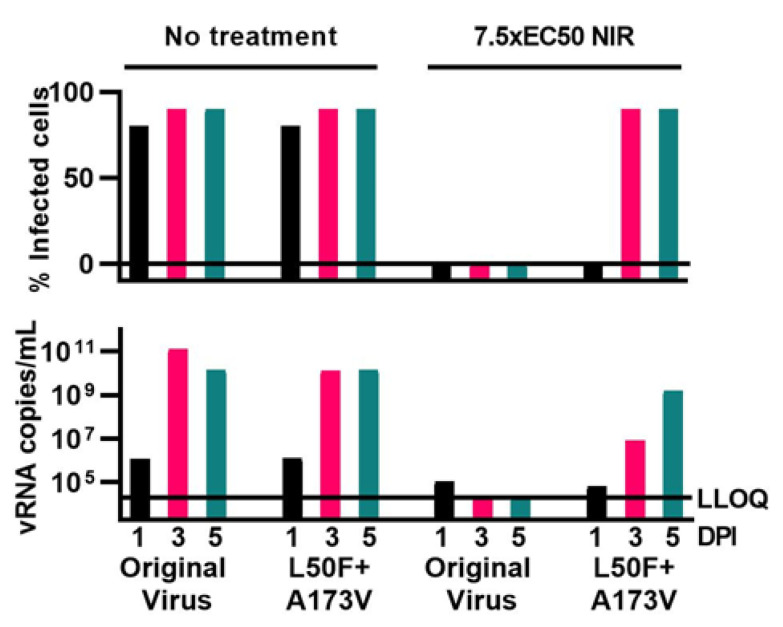
Longer-term treatment of engineered SARS-CoV-2 mutants with nirmatrelvir. VeroE6 cells that were infected with the engineered L50F + A173V double mutant or the original SARS-CoV-2 were treated longer term with the specified fold-EC50 of nirmatrelvir (NIR). % SARS-CoV-2 infected culture cells were determined by immunostaining of spike relative to immunostaining of nuclei on the specified days post-infection (DPI). Viral RNA (vRNA) titers in culture supernatants on the specified DPI were determined by RT-qPCR. LLOQ, lower limit of quantification. For the original SARS-CoV-2, these data have been extracted from a previous publication [36].

The identified putative resistance substitutions L50F and A173V were characterized using a reverse genetics system that reflected the sequence of the original SARS-CoV-2 used in the escape experiments [55]. Thus, we engineered SARS-CoV-2 mutants harboring L50F and A173V, singly and in combination. In short-term concentration–response treatments, L50F and A173V singly conferred only small changes in boceprevir sensitivity (up to 2.0- and 1.4-fold decrease in sensitivity (*p* < 0.0001), respectively). The double mutant showed a 1.8-fold decreased boceprevir sensitivity in VeroE6 and a 2.6-fold decreased sensitivity in A549-hACE2 cells (*p* < 0.0001) (Figure 3, Supplementary Table S2). These substitutions had a somewhat greater impact on viral sensitivity to nirmatrelvir. L50F and A173V singly both conferred a decrease in nirmatrelvir sensitivity of up to 2.3-fold (*p* < 0.0001), while the double mutant showed a 3.5- and 2.1-fold decrease in sensitivity in VeroE6 and A549-hACE2 cells (*p* < 0.0001), respectively. Resistance of the L50F + A173V double mutant to nirmatrelvir was confirmed in longer-term treatments, as this mutant spread under treatment with 7.5-fold EC50 of nirmatrelvir while the original virus was fully suppressed (Figure 4). Thus, the L50F + A173V double mutant showed resistance to nirmatrelvir. Identification of L50F + A173V as nirmatrelvir RAS contributes to a comprehensive overview of RAS mediating resistance to this clinically relevant drug.

### 3.4. Fitness of Mutants with Engineered Mpro Substitutions

**Figure 5:**
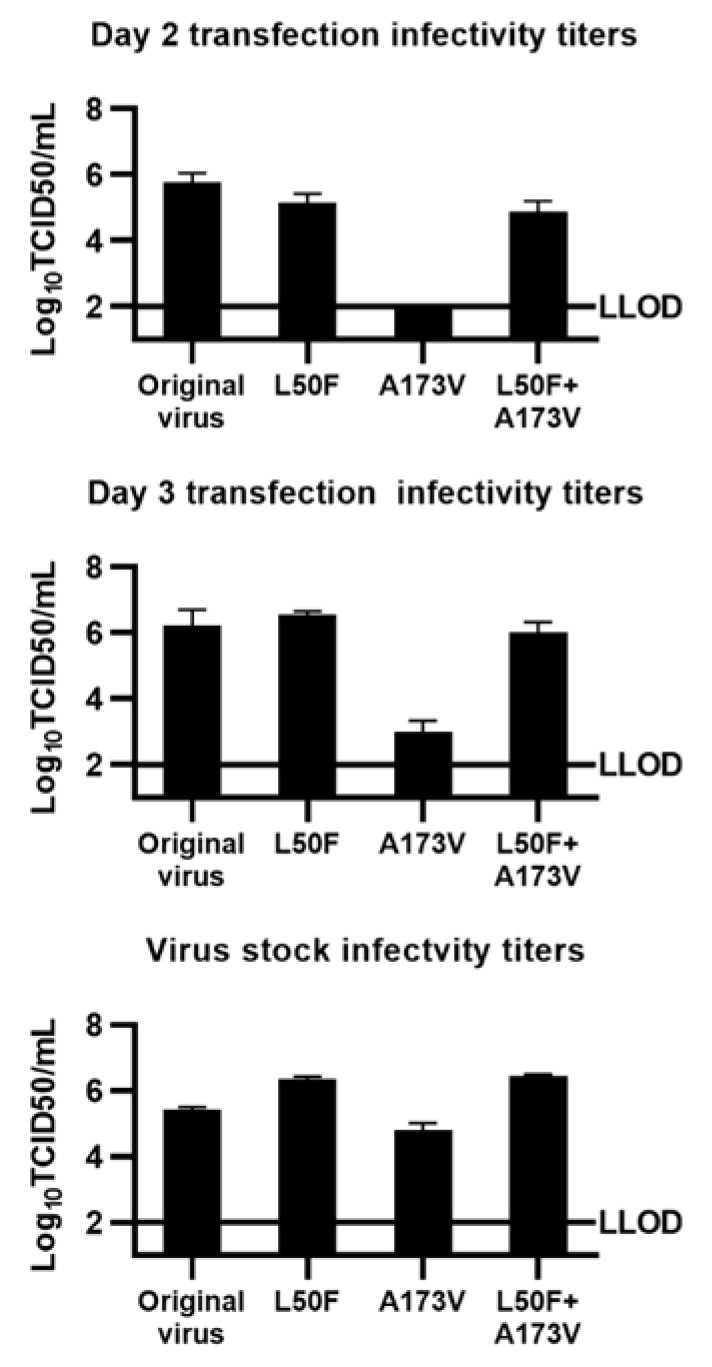
Infectivity titers of engineered SARS-CoV-2 mutants. Viral infectivity titers of supernatants derived from VeroE6 cell cultures infected with SARS-CoV-2 mutants with specified engineered RAS or the original virus. Cultures were either transfected with RNA transcripts or infected with supernatant from first viral passage cultures. As an exception, for infection with the A173V mutant, supernatant from the transfection experiment was applied. Supernatants for determination of infectivity titers were obtained on Days 2 and 3 post-transfection, while virus stocks were supernatant derived at the peak of infection of the respective first or second passage cultures. Datapoints represent 50% tissue culture-infectious doses (TCID50) per mL and are given as means of 3 replicates with SEM. LLOD, lower limit of detection. For the original SARS-CoV-2 and L50F, these data have been published previously [36]. Infectivity titers shown in one graph are determined in the same experiment.

Next, we investigated the impact of the identified substitutions on viral fitness. We demonstrated high viral fitness for the engineered L50F + A173V double mutant and the L50F single mutant, as they showed high infectivity titers, comparable to those of the original virus, in both transfection and viral passage cultures (Figure 5). In contrast, the A173V single mutant showed markedly reduced fitness with viral infectivity titers close to or below the LLOD in transfection cultures. For viral passage cultures, recorded peak infectivity titers showed smaller differences. In addition, we carried out an analysis of genetic stability of these engineered mutants during a serial passage without drug pressure by sequencing analysis. The double mutant and the L50F mutant maintained the engineered substitutions in Mpro during four consecutive viral passages (Supplementary Table S3). In contrast, in the A173V mutant, the engineered substitution had partially reverted after four viral passages. None of the mutants acquired additional substitutions in Mpro during four viral passages (Supplementary Table S3). Moreover, in one independent experiment, the A173V mutant acquired L50F in 33% of viral genomes in the second viral passage. Thus, L50F compensated for the fitness cost of A173V. The L50F + A173V double mutant had high fitness, which could facilitate its emergence, spread, and persistence in populations.

### 3.5. Contribution of Additional Mpro Substitutions to Viral Resistance and Fitness

**Figure 6:**
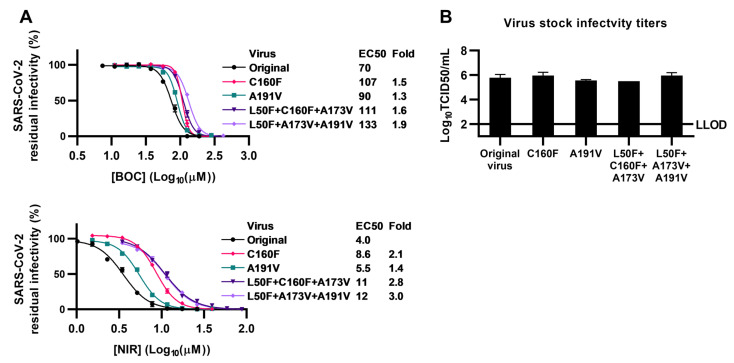
Short-term concentration–response treatments and infectivity titers of engineered SARS-CoV-2 mutants with additional Mpro substitutions. (**A**) VeroE6 cells that were infected with SARS-CoV-2 engineered with specified RAS or original SARS-CoV-2 and that were treated with specified concentrations of boceprevir (BOC) or nirmatrelvir (NIR) were visualized by immunostaining of spike protein and counted automatically. Data points represent residual infectivity determined as count of infected cells in treated infected wells relative to the mean of counts of non-treated infected controls. Data points are given as means of 4 or 7 replicates with SEM. Representative curves and 50% effective concentration (EC50) values from 1 to 13 replicate experiments were determined in GraphPad Prism 8.0.0. In the figure, one representative curve is shown, while EC50 values are median EC50 values calculated based on replicate experiments. Fold resistance values were calculated as EC50_Mutant_/EC50_Original_. Median EC50 values, fold resistance values, and *p*-values are summarized in Supplementary Table S2. For the original SARS-CoV-2 treated with nirmatrelvir, the curves have been presented in a previous publication, while median EC50 values have been calculated in a different manner [36]. (**B**) Viral infectivity titers of supernatants derived at the peak of infection from VeroE6 cell cultures infected with SARS-CoV-2 mutants with specified engineered RAS. Cultures were infected with supernatant derived at the peak of infection from first viral passage cultures. Datapoints represent 50% tissue culture-infectious doses (TCID50) per mL and are given as means of 3 replicates with SEM. LLOD, lower limit of detection. For the original SARS-CoV-2, these data have been published previously [36]. Infectivity titers shown are determined in the same experiment.

During selection of polyclonal escape viruses, sequencing analysis revealed that BOC-EV1 had acquired the additional Mpro substitutions C160F and A191V in 48% and 20% of viral genomes, respectively (Supplementary Table S1). Linkage analysis further revealed that C160F and A191V were not present on the same viral genomes, but individually in combination with L50F + A173V. To investigate the impact of these additional substitutions they were engineered singly and in combination with L50F + A173V. In short-term concentration–response treatments using boceprevir and nirmatrelvir, triple mutants L50F + A173V + C160F or L50F + A173V + A191V showed a decrease in susceptibility to both drugs that was comparable to that of the L50F + A173V double mutant (Figure 6). Similarly, in TCID50 assays, the triple mutants showed infectivity titers that were comparable to that of the L50F + A173V double mutant. When engineered singly, C160F or A191V did not show an impact on resistance or fitness of SARS-CoV-2. Thus, the Mpro substitutions C160F and A191V had no obvious impact on viral resistance or fitness using the applied assays.

### 3.6. Natural Occurrence of Identified Mpro Substitution

**Figure 7:**
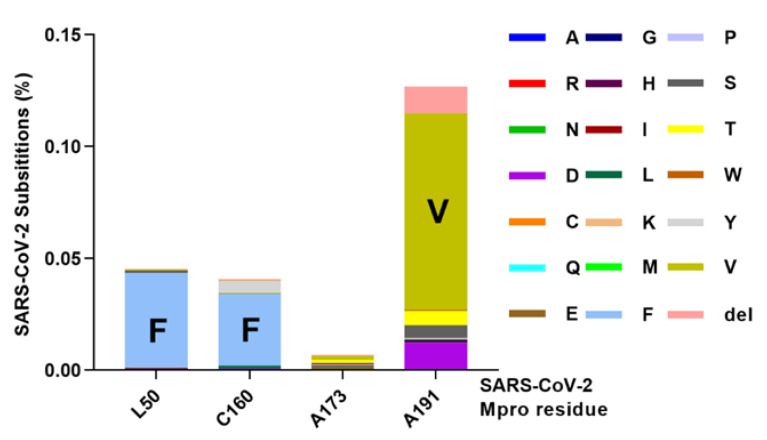
Naturally occurring variations in Mpro at amino acid positions 50, 160, 173, and 191. Datapoints represent % of viruses identified in the GISAID database with specified amino acid substitutions at the specified Mpro positions. del, deletion.

To assess the likelihood of emergence of the identified RAS we investigated if they are naturally occurring. Analysis of sequences extracted from the GISAID database prior to the widespread use of nirmatrelvir in the clinic showed that Mpro positions 50, 160, 173, and 191 were largely conserved but showed some degree of natural variation (Figure 7, Supplementary Table S4). Specifically, substitutions L50F, C160F, and A191V were found at comparable frequencies of 0.04%, 0.03%, and 0.09%, while substitution A173V was found at 0.002%. Thus, all identified Mpro substitutions including L50F and A173V were naturally occurring, even though at a low percentage. The natural occurrence of Mpro substitutions increases the likelihood of their spread in populations, considering a more widespread treatment with nirmatrelvir in the future.

### 3.7. Predicted Influence of Identified Mpro Substitutions on Mpro Structure

**Figure 8:**
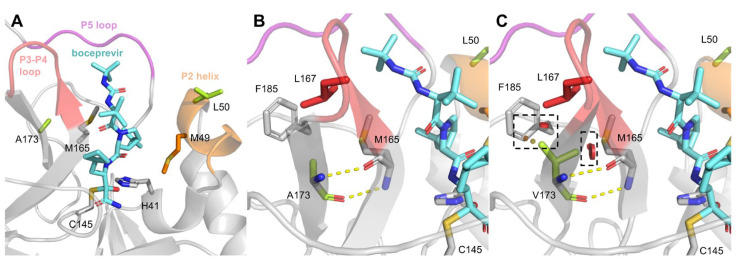
Structural overview and analysis of L50F and A173V in the Mpro-boceprevir structure (PDB entry: 7k40 [59]). (**A**) Figure showing the position of L50 and A173 (limegreen) in the active site of the Mpro-boceprevir structure. The structural elements P2 helix (orange), P3-P4 loop (red), and P5 loop (pink) are highlighted, and boceprevir is shown in an aquamarine stick representation. The catalytic residues (H41 and C145) and the methionines (M49 and M165) are shown as sticks. L50 and A173 are positioned on opposite sides of boceprevir in proximity to M49 and M165, respectively. (**B**) Interactions of A173 in the Mpro structure. The backbone of A173 forms hydrogen bonds with M165 (yellow dashes), whereas the side chain forms hydrophobic interactions with M165, L167, and F185. (**C**) Modeling of the substitution A173V predicts steric clashes (red discs highlighted by black boxes) between the sidechain of V173 and the surrounding residues M165, L167, and F185.

To provide a structural explanation for the observed resistance we carried out an analysis of the structural consequences of the identified RAS. Structural analysis revealed that in the Mpro structure, L50 and A173 are positioned on opposite sides of the S2 subsite of the active site defined by M49 and M165 [61] (Figure 8A). The backbone of A173 forms hydrogen bonds to the backbone of M165, and the sidechain is buried in a hydrophobic environment (Figure 8B). The A173V mutation introduces a bulkier sidechain that, as suggested by in silico mutagenesis, will lead to steric clashes with M165, L167, and F185 (Figure 8C). Thus, structural Mpro analysis suggested that A173V resulted in changes in the Mpro active site. Changes in the Mpro active site induced by A173V are likely perturbing its interaction with Mpro inhibitors, resulting in the observed resistance.

## 4. Discussion

In this study, we identified nirmatrelvir RAS L50F and A173V based on resistance selection during boceprevir escape experiments using an infectious SARS-CoV-2 cell culture system. While A173V was the main driver of resistance, L50F compensated for the fitness cost of A173V. Thus, the identified L50F + A173V escape viruses showed resistance and high fitness in our in vitro model. Importantly, this study refines the resistance profile of the first-in-line compound for treatment of COVID-19, nirmatrelvir. It is highly relevant to work towards a comprehensive overview of Mpro inhibitor RAS, which goes beyond the findings from initial proof-of-concept studies [36,37,38,39,44], as current COVID-19 treatments rely on the Mpro inhibitor nirmatrelvir and as additional protease inhibitors are being developed.

To our knowledge, by now, SARS-CoV-2 escape from boceprevir has not been reported. Even though boceprevir has limited clinical potential for treatment of COVID-19, it is relevant to study boceprevir escape because of the structural similarities between boceprevir and nirmatrelvir, and additional Mpro inhibitors in clinical development. Thus, as exemplified in this study, resistance profiles can potentially overlap among structurally related Mpro inhibitors, and therefore, boceprevir escape studies can help identify novel RAS of clinical relevance.

Due to its cytotoxicity, we could apply only relatively low concentrations of boceprevir. This likely explains why A173V was selected, which only conferred a relatively low degree of resistance compared to substitutions at position 166, reported by us and others to confer high resistance to nirmatrelvir [36,37,38,39,44]; the effect of RAS at position 166 on boceprevir resistance remains to be investigated in the future. Interestingly, the two polyclonal escape viruses showed somewhat higher resistance than the engineered L50F + A173V mutant. Such small discrepancies in phenotype for recombinant mutants versus polyclonal escape viruses have been observed for other viruses and inhibitors and might be due to substitutions outside the drug target contributing to resistance, either directly or indirectly via enhanced fitness [62,63].

Underlining the relevance of A173V as a nirmatrelvir RAS, a previous study identified A173V in SARS-CoV-2 selected during nirmatrelvir escape experiments in cell culture. Reverse genetics in an infectious culture system revealed that A173V conferred a small (1.8-fold) decrease in nirmatrelvir susceptibility in VeroE6 cells. While the combination of A173V with T21I or T21I + T304I resulted in a 3.1- and 15-fold decrease in nirmatrelvir susceptibility, respectively, the combination of A173V with L50F was not investigated [37]. Another study investigated naturally occurring Mpro polymorphisms in an infectious culture system. Here, the authors reported an 8.1-fold decrease in nirmatrelvir susceptibility for A173V and a 62.5-fold decrease for ΔP168 + A173V, while ΔP168 alone did not confer resistance [44]. In line with these findings, we described that culture-infectious SARS-CoV-2 escape viruses and engineered mutants with A173V have decreased nirmatrelvir susceptibility. In addition, we investigated the L50F + A173V double mutant, which has not been studied before by reverse genetics. Importantly, we confirmed that A173V together with L50F mediated resistance in longer-term treatments. We have previously shown that small EC50 differences in short-term treatment assays can translate into bigger differences in longer-term treatments for HCV protease inhibitor RAS, which had been associated with clinical resistance [64]. The maximum serum concentration (Cmax)/EC50 ratio for nirmatrelvir is 1.1 and 55 for the original SARS-CoV-2 in VeroE6 and A549-hACE2 cells, respectively, based on EC50 determined in this and a previous study [36]. The Cmax/EC50 ratio was reduced to 0.3 and 22 for the engineered L50F + A173V mutant and to 0.1 and 8.8 for BOC-EV1, the most resistant of the two polyclonal escape viruses, in VeroE6 and A549-hACE2 cells, respectively. While such a reduction could have an impact on clinical efficacy, the clinical relevance of these substitutions needs to be determined in future research. While certain substitutions in Mpro, including E166V, have been detected in patients following nirmatrelvir treatment, A173V has so far not been detected [21].

Together with our previous findings where we showed that L50F compensated for the fitness cost of E166V [36], our current data suggest that L50F has a broader fitness compensating effect for Mpro resistance substitutions. Importantly, we have shown that the pre-existence of naturally occurring L50F facilitated the selection of nirmatrelvir-resistant SARS-CoV-2 escape viruses with additional Mpro substitutions [36]. Thus, the background frequency of L50F and A173V, as recorded in the GISAID database as well as the high fitness of L50F + A173V, increase the risk of emergence and subsequent spread of Mpro inhibitor-resistant variants within populations. The emergence of Mpro inhibitor-resistant variants could potentially jeopardize the availability of effective treatment options for patients with severe COVID-19.

In one escape experiment, we identified Mpro substitutions C160F or A191V, selected in addition to L50F + A173V. Further reverse genetics analysis showed that C160F or A191V did not contribute to resistance in short-term treatment assays or viral fitness in TCID50 assays. These substitutions might be co-selected polymorphisms; however, A191V also occurred in the fifth escape experiment, even though on different genomes than A173V. It might be more likely that these substitutions slightly contribute to resistance or fitness to a level that cannot be detected in the applied assays.

L50F and A173V are located at structural elements that are important for the conformational flexibility of the active site, respectively, in the P2 helix and near the P3-P4 loop (Figure 8A). This conformational flexibility determines which substrates/inhibitors can bind to Mpro [48]. We hypothesize that the resistance observed for A173V against boceprevir and nirmatrelvir can be explained by the steric clashes (Figure 8C), which may induce changes in the conformational dynamics of the active site region, leading to a less favorable binding of boceprevir and nirmatrelvir. More specifically, we hypothesize that the steric clashes may change the interactions between the active site methionines (M49 and M165) and the inhibitors. This is supported for nirmatrelvir by molecular dynamics simulation (MDS) results of Mpro-nirmatrelvir showing that A173V increases the flexibility of M49 and the P2 helix [44]. The lower fitness of the A173V mutant could possibly be explained by A173V also leading to less favorable substrate binding. Considering the location of L50 in the P2 helix and its proximity to M49, the fitness compensatory effect of L50F might be explained by L50F + A173V compensating the conformational changes in the P2 helix induced by A173 and thereby improving substrate binding while maintaining reduced affinity for the inhibitors.

Future research should focus on further refinement of resistance profiles for Mpro inhibitors such as nirmatrelvir and ensitrelvir, also investigating cross-resistance. To this end, it will be relevant to carry out MDS analyses to assess the structural consequences of RAS on interactions of Mpro with relevant inhibitors and with its natural substrates.

A drawback of the study is that it is in vitro only, which does not fully reflect in vivo conditions, and thus, it will be important to further investigate the clinical relevance of identified RAS. Resistant Mpro variants have been shown to be transmissible in hamsters [65] and have already been found in patients. However, more systematic studies are needed to associate treatment failure with the identified RAS. Thus, for patients who experience treatment failure, it will be relevant to carry out sequence analysis to investigate if they are infected with SARS-CoV-2 variants that harbor putative RAS. Finally, it will be important to develop Mpro inhibitors with efficacy against SARS-CoV-2 variants with so far described nirmatrelvir RAS, by screening of drug libraries or by optimization of current Mpro inhibitors.

In conclusion, we report highly fit SARS-CoV-2 mutants with substitutions L50F and A173V in Mpro that confer decreased susceptibility to the oral SARS-CoV-2 Mpro inhibitor, nirmatrelvir. This study contributes to refining the resistance profile of nirmatrelvir, the first-in-line COVID-19 treatment, and the identified RAS could be included in resistance testing of emerging Mpro inhibitors. Characterization of Mpro inhibitor resistance profiles facilitates population monitoring to ensure the availability of safe and efficient treatments for patients suffering from COVID-19. Further, as Mpro is highly conserved between different coronaviruses, the definition of Mpro inhibitor resistance profiles contributes to pandemic preparedness.

## Data Availability

All relevant data are included in the manuscript.

## Supplementary Materials

The following supporting information can be downloaded at https://www.mdpi.com/article/10.3390/v15091970/s1, Supplementary Table S1: Non-synonymous mutations in boceprevir escape viruses localizing to sequences encoding Mpro; Supplementary Table S2: Potency of boceprevir and nirmatrelvir against original SARS-CoV-2 and SARS-CoV-2 mutants; Supplementary Table S3: Non-synonymous mutations in serially passaged SARS-CoV-2 mutants in the complete open reading frame (ORF); Supplementary Table S4: Naturally occurring substitutions in SARS-CoV-2 Mpro.
