# Supplementary material for: Substitutions in SARS-CoV-2 Mpro Selected by Protease Inhibitor Boceprevir Confer Resistance to Nirmatrelvir
Source: Viruses. 2023 Sep 21;15(9):1970. doi: 10.3390/v15091970 (PMC10536901; doi:10.3390/v15091970)
Supplement: Supplementary file 1 [file viruses-15-01970-s001.zip › viruses-2562685-supplementary materials.pdf]

Supplementary Table S1. Non-synonymous mutations in boceprevir escape viruses localizing to sequences encoding Mpro.

Nucleotide change[g]	Amino acid change[g]	SARS-CoV-2 protein[h]	Escape 1[a] - %[b]			Escape 2[c] - %[b]		Escape 3[d] - %[b]	Escape 4[e] - %[b]		Escape 5[f] - %[b]
			5xEC50 D16[i]	5xEC50 D57[i]	5xEC50 D74[i]	5xEC50 D24[i]	7xEC50 D57[i]	7xEC50 D124[i]	7xEC50 D24[i]	7xEC50 D57[i]	7xEC50 P3D5[i]
C10116T	T21I	Mpro	-	-	-	16	-	92	-	-	16
C10202T	L50F	Mpro	-	100	100	81	100	100	-	100	-
G10533T	C160F	Mpro	-	-	48	-	-	-	-	-	-
C10572T	A173V	Mpro	-	98	100	81	100	100	98	100	78
C10626T	A191V	Mpro	-	-	20	-	-	-	-	100	14
A10842G	D263G	Mpro	-	-	-	-	-	-	-	-	48

[a] Escape 1, source of polyclonal escape virus BOC-EV1: Continuous virus culture in VeroE6 cells for 74 days under treatment with boceprevir. Treatment concentration was increased when virus had spread under a given concentration. At the timepoint when NGS was carried out the virus had spread to ≥70% of culture cells The culture was treated with 5xEC50 boceprevir from day 3 to 24 post infection, with 5xEC50 from day 28 to 36, with 4xEC50 from day 37 to 42 and with 5xEC50 from day 43 to 74. In the given time intervals, treatment was carried out every 2-3 days.

[b] Frequency (%) of non-synonymous nucleotide changes in the viral genome sequences encoding Mpro recorded by NGS. Analyzed viruses were harvested from cell culture supernatant. Changes that occurred with at least 10% frequency in at least one of the analyzed virus populations were included in this table. -, frequency of the given change was <10%.

[c] Escape 2, source of polyclonal escape virus BOC-EV2: Continuous virus culture in VeroE6 cells for 57 days under treatment with boceprevir. Treatment concentration was increased when virus had spread under a given concentration. At the timepoint when NGS was carried out the virus had spread to ≥70% of culture cells. The culture was treated with 5xEC50 boceprevir from day 2 to 53 post infection and with 7xEC50 from day 54 to 66. In the given time intervals, treatment was carried out every 2-3 days.

[d] Escape 3: Continuous virus culture in VeroE6 cells for 124 days under treatment with boceprevir.
Treatment concentration was increased when virus had spread under a given concentration. At the timepoint when NGS was carried out the virus had spread to ≥70% of culture cells. The culture was treated directly after infection with 2.5xEC50 boceprevir for 21 days, with 3xEC50 from day 22 to 27, with 4xEC50 from day 28 to 33, with 5xEC50 from day 34 to 100 and with 7xEC50 from day 101 to 124. In the given time intervals, treatment was carried out every 2-3 days.

[e] Escape 4: Continuous virus culture in VeroE6 cells for 57 days under treatment with boceprevir. Treatment concentration was increased when virus had spread under a given concentration. At the timepoint when NGS was carried out the virus had spread to ≥40% of culture cells. The culture was treated with 7xEC50 boceprevir from day 2 to 57 post infection. In the given time interval, treatment was carried out every 2-3 days.

[f] Escape 5: Primary escape culture followed by 3 viral passages in VeroE6 cells under treatment with boceprevir. Treatment concentration was increased when virus had spread under a given concentration. At the timepoint when NGS was carried out the virus had spread to ≥90% of culture cells. All cultures were treated directly after infection and then every 2-3 days. The primary escape culture was treated with 2.5xEC50 boceprevir for 21 days, with 3xEC50 from day 22 to 27 and with 4xEC50 from day 28 to 34. The 1st passage (P1) culture was treated with 5xEC50 for 3 days. The 2nd passage (P2) culture was treated with 7xEC50 for 3 days. The 3rd passage (P3) culture was treated with 7xEC50 for 5 days.

[g] Nucleotide / amino acid position numbers and original nucleotides / amino acids, given in front of the position numbers, relate to the nucleotide / amino acid sequence of the SARS-CoV-2/human/Denmark/DK-AHH1/2020 strain (GenBank accession number MZ049597). The changed nucleotides / amino acids, acquired during continuous virus culture or passage under boceprevir treatment, are given after the position numbers.

[h] SARS-CoV-2 protein, to which the identified amino acid changes located relating to the SARS-CoV-2/human/Denmark/DK-AHH1/2020 strain (GenBank accession number MZ049597).

[I] Specification of conditions under which viral genomes subjected to NGS were sampled. Fold EC50 applied and passage (P) and/or day (D) post infection at sampling time.

Supplementary Table S2. Potency of boceprevir and nirmatrelvir against original SARS-CoV-2 and SARS-CoV-2 mutants.

**Boceprevir VeroE6**	**EC50 (μM)**[a]	**Fold**[b]	***P*-value**[c]	**Exp**[d]
**Virus**				
original virus	70	-	-	3
BOV-EV1	327	4.7	< 0.0001	1
BOC-EV2	236	3.4	< 0.0001	1
L50F	99	1.4	< 0.0001	2
C160F	107	1.5	< 0.0001	1
A173V	98	1.4	< 0.0001	2
A191V	90	1.3	< 0.0001	1
L50F+A173V	125	1.8	< 0.0001	3
L50F+C160F+A173V	111	1.6	< 0.0001	1
L50F+A173V+A191V	133	1.9	< 0.0001	1
**Boceprevir A549-hACE-2**	**EC50 (μM)**[a]	**Fold**[b]	***P*-value**[c]	**Exp**[d]
**Virus**				
original virus	24	-	-	3
BOV-EV1	67	2.8	< 0.0001	3
BOC-EV2	94	3.9	< 0.0001	3
L50F	48	2.0	< 0.0001	1
A173V	11	0.5	0.4405	1
L50F+A173V	62	2.6	< 0.0001	1
**Nirmatrelvir VeroE6**	**EC50 (μM)**[a]	**Fold**[b]	***P*-value**[c]	**Exp**[d]
**Virus**				
original virus	4.0	-	-	13
BOV-EV1	30	7.3	< 0.0001	1
BOC-EV2	25	6.2	< 0.0001	1
L50F	9.3	2.3	< 0.0001	1
C160F	8.6	2.1	< 0.0001	1
A173V	7.4	1.8	< 0.0001	1
A191V	5.5	1.4	< 0.0001	1
L50F+A173V	14	3.5	< 0.0001	2
L50F+C160F+A173V	11	2.8	< 0.0001	2
L50F+A173V+A191V	12	3.0	< 0.0001	2
**Nirmatrelvir A549-hACE-2**	**EC50 (μM)**[a]	**Fold**[b]	***P*-value**[c]	**Exp**[d]
**Virus**				
original virus	0.08	-	-	7
BOV-EV1	0.5	6.1	< 0.0001	3
BOC-EV2	0.6	6.6	< 0.0001	3
L50F	0.1	1.2	0.0983	2
A173V	0.2	2.3	0.0001	1
L50F+A173V	0.2	2.1	< 0.0001	3

[a] EC50, half maximal effective concentration of the specified inhibitor against the specific virus.

[b] Fold resistance values were calculated as $EC50_{\text{Escape Virus}}/EC50_{\text{Original}}$

[c] *P*-values.

[d] Number of replicate experiments. Curves derived from representative experiments are shown in Figure 1, 3 and 6.

Supplementary Table S3. Non-synonymous mutations in serially passaged SARS-CoV-2 mutants in the complete open reading frame (ORF).

**Nucleotide change**[b]	**Amino acid change**[b]	**SARS-CoV-2 protein**[c]	**SARS-CoV-2 mutants-%**[a] **L50F P4D2**[d]	**A173V P4D2**[d]	**L50F+A173V P4D2**[d]
C10202T	L50F	Mpro	100	-	100
C10572T	A173V	Mpro	-	89	100
C11379T	A136V	nsp6	-	90	-
C19325T	P429L	nsp14	-	12	-
A22296G	H245R	S	28	-	-
C23525T	H655Y	S	11	-	-
G23607A	R682Q	S	-	-	87
C26309A	A22D	E	-	30	-

[a] Frequency (%) of non-synonymous nucleotide changes in the complete ORF of the specified SARS-CoV-2 mutants following 4 viral passages in cell culture, as recorded by NGS. Analyzed viruses were harvested from cell culture supernatant. Changes that occurred with at least 10% frequency in at least one of the analyzed virus populations were included in this table. -, frequency of the given change was <10%.

[b] Nucleotide / amino acid position numbers and original nucleotides / amino acids, given in front of the position numbers, relate to the nucleotide / amino acid sequence of the SARS-CoV-2/human/Denmark/DK-AHH1/2020 strain (GenBank accession number MZ049597). The changed nucleotides / amino acids, acquired following serial passage, are given after the position numbers.

[c] SARS-CoV-2 protein, to which the identified amino acid changes located relating to the SARS-CoV-2/human/Denmark/DK-AHH1/2020 strain (GenBank accession number MZ049597).

[d] Specification of the SARS-CoV-2 mutant and the time when viral genomes subjected to NGS were sampled, being passage 4 (P4) day 2 (D2) post infection for all mutants.

Supplementary Table S4. Naturally occurring substitutions in SARS-CoV-2 Mpro.

	Amino acid residue[b]	SARS-CoV-2 Mpro residue[a] L50	C160	A173	A191
Number of viruses[c] (Total: 10,302,924 viruses)	A	3	1	0	0
	R	20	2	79	13
	N	0	9	66	8
	D	27	63	77	1248
	C	1	0	3	5
	Q	0	0	3	11
	E	0	1	1	7
	G	2	3	3	79
	H	13	0	0	11
	I	38	5	2	5
	L	0	116	13	29
	K	11	2	2	48
	M	1	2	1	8
	F	4370	3306	2	10
	P	11	1	7	10
	S	97	35	82	582
	T	2	3	122	655
	W	0	4	4	51
	Y	0	563	1	2
	V	39	18	181	9043
	del	26	63	62	1246
	Number of substitutions[d]	4650	4195	709	13023

[a] SARS-CoV-2 Mpro residues of interest due to identification of RAS in this study.
[b] Amino acid residues using one letter codes; del, deletion.
[c] For this analysis, a total of 10,302,924 SARS-CoV-2 sequences were retrieved from the GISAID database on April 18th, 2022, prior to widespread use of nirmatrelvir in patients.
[d] Total number of sequences with any substitution at L50, C160, A173 or A191 in Mpro.
